# Distribution of *Giardia duodenalis* Assemblages A and B among Children Living in a Remote Indigenous Community of the Northern Territory, Australia

**DOI:** 10.1371/journal.pone.0112058

**Published:** 2014-11-20

**Authors:** Amy J. Asher, Deborah C. Holt, Ross M. Andrews, Michelle L. Power

**Affiliations:** 1 Department of Biological Sciences, Macquarie University, Sydney, New South Wales, Australia; 2 Menzies School of Health Research, Charles Darwin University, Casuarina, Northern Territory, Australia; University of Camerino, Italy

## Abstract

Giardiasis is a communicable gastrointestinal disease caused by *Giardia duodenalis* and two genetic assemblages, A and B, cause human infection. In remote Indigenous communities of Australia, giardiasis is highly prevalent among children but disease transmission is poorly understood. This study investigated the prevalence of *Giardia* and genetic subtypes contributing to human disease in a remote Indigenous community, in the Northern Territory of Australia. Eighty-seven faecal samples were collected from 74 children (<15 years) over an 18 month period, and the distribution of positive cases relative to participant age and gender were examined. Screening by microscopy and 18S rRNA PCR amplification showed 66.7% (58/87) of faecal samples were positive for *Giardia*. Both males and females were equally affected and high detection rates were obtained for participants aged 0–<5 years and 5–<10 years (66.0 and 60.0% respectively). For 58.6% of the positive samples, *Giardia* was only detected by 18S rRNA PCR. Approximately 75% of cases were assemblage B, and subassemblage analyses using terminal restriction fragment length polymorphism of the glutamate dehydrogenase gene demonstrated that a variety of genetic variants were present. The high proportion of positive cases that were not detectable by microscopy, and dominance of assemblage B cases highlights the need for further research in this community, to assess the contribution of *Giardia* to chronic gastrointestinal disease among children, and to understand conditions conductive to assemblage B transmission.

## Introduction

In Australia, the mortality rates for Indigenous children (0–14 years) are more than two times higher than the rates for non-Indigenous children, and overall life expectancy of Indigenous Australians remains 9.7–11.5 years below the non-Indigenous population [1]. In remote Indigenous communities, high rates of gastrointestinal, respiratory, and skin infections are common and children are most at risk from constant infection and re-infection [2]. Chronic childhood infections are linked to poor physical and cognitive development, poor educational outcomes, socio-economic disadvantage, and poor health status throughout life [3].

*Giardia duodenalis*, a protozoan parasite, is a significant cause of gastrointestinal disease (giardiasis) and morbidity [4]. In remote Indigenous communities giardiasis prevalence is high, ranging from 15 to 36% [4], [5], compared to a national prevalence of 2 to 7% [6]. Among Indigenous children living in remote communities, prevalence of giardiasis is estimated between 32 to 65% and frequency of transmission is comparable to rates observed in developing nations [4], [7], [8]. Constant exposure to *Giardia* leads to chronic gastrointestinal disease, malnutrition, and failure to thrive [9].

In the Northern Territory of Australia approximately 80% of the Indigenous population live in areas classified as remote or very remote [1], [10]. Communities range in size from small groups to a few thousand people, and communities are geographically isolated [10]. Overcrowded living conditions, inadequate housing and community sanitation facilities, and poor personal hygiene contribute to the high rates of disease transmission in these communities [10]. Previous initiatives to manage infectious diseases have included improved housing and community wide drug treatment programs; however, many diseases continue to persist with high infection rates [2], [3]. The high frequency of *Giardia* transmission and continued persistence in remote Indigenous communities is poorly understood.

*Giardia duodenalis* is a species complex and two genetic assemblages (A and B) infect humans, domestic animals, and wildlife [11], [12]. These assemblages are broad clusters of genetically related isolates [6] and four human infective subassemblages (AI, AII, BIII and BIV) have been previously described [13], [14]. Genetic diversity within assemblage B, however, is higher than assemblage A; assemblage B subgroups are unresolved; and numerous assemblage B genotypes contribute to human and animal infection [13], [15]. Identification of different genetic types that contribute to disease enables differences in host specificities, transmission cycles, and sources of infection to be more closely examined [15].

Several epidemiological studies of giardiasis have been conducted in remote Indigenous communities in Australia [4], [7], [8], but few have performed molecular analyses [16], [17] to identify genetic subtypes contributing to high infection rates. The few molecular epidemiological studies that have been undertaken predate the current understanding of *Giardia*, generated by molecular methods [16], [17]. Subtype identification requires analyses of *G. duodenalis* DNA and are not conducted in routine pathology screenings. The geographic remoteness of communities limits access and feasibility of performing offsite DNA screening for samples. Additionally, in the Northern Territory giardiasis is not listed as a notifiable disease and epidemiological information is not routinely collected for positive cases. It is unclear if different *G. duodenalis* genetic variants exist in communities and contribute to high reinfections rates among children [8].

The purpose of this study was to investigate the prevalence of *G. duodenalis* among children in a remote Indigenous Australian community, and to examine the distribution of genetic assemblages and diversity of subtypes present. Knowledge of *G. duodenalis* assemblages and subtypes is required to understand disease transmission, assess public health risks posed by community conditions, and to understand how these factors contribute to persistent giardiasis infections in these communities.

## Materials and Methods

### Ethics statement

Ethical approval for the community based mass drug administration trial, including the collection and analysis of faecal samples, was obtained from the Human Research Ethics Committee of the Menzies School of Health Research and Northern Territory Department of Health. Additional approval for the analysis of the faecal samples was also obtained from the Macquarie University Human Research Ethics Committee. Written informed consent was obtained from the parent or guardian for the collection and analysis of faecal samples from children. Participant age group and gender were used to analyse the distribution of *Giardia* positive cases. No additional or identifying information was provided for participants included in this study.

### Faecal samples, microscopy and DNA extraction

To investigate the prevalence of *G. duodenalis*, we examined 87 faecal samples that were collected as part of a mass drug administration trial of ivermectin for the control of scabies and strongyloidiasis in a remote community in the Northern Territory [18]. Faecal samples were acquired from children aged less than 15 years as a routine component of the primary study [18]. A total of 87 faecal samples were collected from 74 children at three times points over an 18 month period. Samples were examined by direct smear for the presence of *Giardia* and the remainder stored in ethanol at −20°C until DNA extraction. DNA was extracted from 200 mg of faeces using a PowerSoil DNA Isolation Kit (Mo Bio, Calsbad, CA) according to the manufacturer’s instructions.

### PCR and sequence analyses of the 18S rRNA gene

DNA samples were screened for the presence of *Giardia* DNA by PCR amplification of a fragment of the small subunit ribosomal RNA (18S rRNA) gene, using a two-step nested PCR method and cycling conditions described [17], [19] with primers RH11, RH4LM, GiAR18SeR and GiAR18SiR. The GC-RICH PCR System, dNTPack (Roche Diagnostics, Indianapolis, IN, USA) was used to prepare PCR reactions as previously described [20], [21], in a total volume of 25 µl, containing 1 µl of template DNA.

DNA sequencing was performed for 18S rRNA PCR products to identify *Giardia duodenalis* assemblages. Positive secondary PCR products were purified with a QIAquick PCR Purification Kit (Qiagen, Melbourne, Australia), according to the manufacturer’s instructions. Purified PCR products were sequenced by Macrogen Inc. (Seoul, Korea) in both the forward and reverse directions using the secondary PCR primers (GiaR18SeR and GiaR18SiR). Geneious PRO version 5.3.6 (Biomatters Ltd, Auckland, New Zealand) was used to generate a contiguous 18S rRNA sequence (174–175 bp) for each sample. To identify *G. duodenalis* assemblages from 18S rRNA contigs, BLASTn sequence searches of the NCBI GenBank database (http://www.ncbi.nlm.gov/genbank/index.html) were performed. 18S rRNA contigs were aligned to previously described 18S rRNA sequences retrieved from the GenBank database (accession numbers AF199446 (assemblage A), AF199447 (assemblage B), AF199449 (assemblage C), AF199443 (assemblage D), AF199448 (assemblage E), AF199444 (assemblage F), AF199450 (assemblage G) [22]. Nucleotide sequence alignments were performed using Clustal W [23] in Geneious PRO. All 18S rRNA contiguous sequences that were generated in this study were submitted to the European Nucleotide Archive (ENA) (http://www.ebi.ac.uk/ena) under accession numbers LN611577–LN611621.

### Terminal-restriction fragment length polymorphism (T-RFLP) for gdh products

PCR amplification of the glutamate dehydrogenase (*gdh*) gene was performed for all samples to enable subassemblage identification. A 432bp fragment of the *gdh* gene was amplified following the semi-nested protocol and cycling conditions described [14] using primers GDHeF, GDHiF and GDHiR. The GC-RICH PCR System, dNTPack was used to prepare the reactions [21] in a total volume of 25 µl, containing 1 µl of template DNA.

To identify *G. duodenalis* subassemblages and examine the population structure of individual infections, *gdh* PCR products were analysed by T-RFLP using the method previously described [24]. Secondary fluorescent PCR was conducted on *gdh* PCR positive samples and products were purified as described above. To generate fluorescent terminal restriction fragments (T-RFs), products were digested with two restriction endonucleases; *Nla IV* (New England BioLabs) and *Rsa I* (Roche Diagnostics, Indianapolis, IN, USA) [14]. Fluorescent T-RFs were detected by capillary electrophoresis at the Macquarie University Sequencing Facility [25]. Length in basepairs of T-RFs were estimated using the DNA size standard LIZ 500 (Applied Biosystems) and GeneScan software version 4.0 (Applied Biosystems) [24], [25].

### Statistical analyses

Differences in *Giardia* positivity and *G. duodenalis* assemblages among males and females, participant age groups, and collection round were investigated using chi-squared statistical analyses. The frequency of males and females among the total number of samples collected were used to determine expected frequencies for *Giardia* positivity, assuming no gender bias for *Giardia* infections, and assuming equal distributions of genetic assemblages. The number of samples collected for participants aged >10 years, and those collected in round 3 were too small to determine the statistical significance of results, and were recorded as observations.

## Results

### Comparison of *Giardia* screening results over the 18 month collection period

Screening for *Giardia* by microscopy and 18S rRNA PCR produced positive results for a total of 58/87 (66.7%) faecal samples collected over the study period (Table 1). A total of 24/87 (27.6%) were positive by microscopy, and 54/87 (62.1%) were positive by 18S rRNA PCR amplification (Table 1). Comparison of results showed that of the 58 positive samples, 20/58 (34.5%) were positive by both screening methods, 34/58 (58.6%) were positive by 18S rRNA PCR only, and 4/58 (6.9%) samples were positive by microscopy only. *Giardia* was not detected by either screening method in 29/87 (33.3%) samples.

**Table 1:** Comparison of *Giardia* positive 18S rRNA PCR results to microscopy screenings by participant age, gender, and collection round for 87 faecal samples (74 participants) collected from children living in a remote Indigenous community in the Northern Territory.

		Positive Faecal Samples (n = 87)				Participant Data (n = 74)			
		Collection round				Age (years)			
	Gender	Round 1	Round 2	Round 3	Total (%)	0–<5	5–<10	10–<15	Total (%)
Collected	Males	19	24	5	48 (55.2)	28	10	0	38 (51.4)
	Females	16	23	0	39 (44.8)	22	10	4	36 (48.6)
	Total	35	47	5	87	50	20	4	74
Microscopy	Males	7	8	2	17 (70.8)	9	4	0	13 (68.4)
	Females	4	3	0	7 (29.2)	4	2	0	6 (31.6)
	Total	11	11	2	24 (27.6)	13	6	0	19 (25.7)
18S rRNA	Males	10	16	5	31 (57.4)	18	6	0	24 (53.3)
	Females	6	17	0	23 (42.6)	15	3	3	21 (46.7)
	Total	16	33	5	54 (62.1)	33	9	3	45 (60.8)
Total positive(combined results)	Males	11	17	5	33 (56.9)	18	7	0	25 (52.1)
	Females	8	17	0	25 (43.1)	15	5	3	23 (47.9)
	Total	19	34	5	58 (66.7)	33	12	3	48 (64.9)

Detection rates of *Giardia* in faecal samples (combined microscopy and PCR results) were high in all three collection periods. At round 1 (month 0), 54.3% (19/35) of samples were positive, 72.3% (34/47) were positive at round 2 (month 12), and all five of the samples collected during a more limited follow-up at round 3 (month 18) were positive (Table 1). The increase in positivity detected between rounds 1 and 2 was almost entirely attributable to an increase in PCR positivity in round 2, compared with round 1. There was no difference in the percentage of positive samples detected by microscopy at the two collection rounds (11/35 (31.4%) versus 11/47 (23.4%) respectively) (Table 1).

### *Giardia* positivity for 74 participants by age, gender and collection round

In total there were 74 participants that contributed the 87 faecal samples over the 18 month collection period. Of the 13 participants that contributed samples twice, 11 contributed samples at rounds 1 and 2 of collection, and two participants contributed samples at rounds 2 and 3 of collection. Only the first sample was included for each participant in the following analyses based on total participants. Participants were aged 0–<5 years (50/74, 67.6%), 5–<10 years (20/74, 27.0%), and 10–<15 years (4/74, 5.4%). There were slightly more males (38/74, (51.4%) than females (36/74, 48.6%) included in the study (Table 1).

The total number of participants with positive screening results for *Giardia* by microscopy and/or 18S PCR was 48/74 (64.9%) (Table 1). There was no difference in the total number of positive males (25/48, 52.1%) and females (23/48, 47.9%), or between those aged 0–<5 years (33/50, 66.0%) and 5–<10 years (12/20, 60.0%). A total of 19/74 (25.7%) participants were positive for *Giardia* by microscopy. There was no difference in microscopy positivity between the two age groups (0–<5 years 13/50, 26.0%; 5–<10 years 6/20, 30.0%); however, more males were positive (13/19, 68.4%) than females (6/19, 25.7%).

A total of 45/74 participants were 18S rRNA PCR positive and there was no difference in the proportion of positive males (24/45, 53.3%) and females (21/45, 46.7%) (Table 1). PCR positivity, however, was 46.7% greater for those aged 0–<5 years (33/50, 66.0% positive) compared with those aged 5–<10 years (9/20, 45.0% positive) (Chi-sq = 7.26, df = 1, p-value = 0.01). For participants aged 0–<5 years, 18S rRNA positivity was higher at collection round 2 (23/30, 76.7%) compared with collection round 1 (14/27,51.9%) (Chi-square = 5.66, df = 1, p-value = 0.02), and this was consistent with the increase in 18S rRNA PCR positivity previously described for round 2. Changes in PCR positivity for participants aged 5–<10 years and 10–<15 years between collection rounds 1 and 2 could not be determined due to small sample numbers within these groups. The equal distributions of 18S rRNA positivity among males and females, however, were maintained between rounds 1 and 2 (Table 1).

### DNA sequence analyses of 18S rRNA amplicons and identification of *Giardia* assemblages A and B

DNA sequencing was successful for 45/54 18S rRNA PCR positive samples, and a complete contiguous sequence for the secondary PCR product was assembled for 43 samples. A shorter (158 bp) contiguous 18S rRNA sequence was assembled for two further samples. Polymorphic nucleotide positions for all *Giardia* assemblages were within this 158 bp region, and thus it was possible to genotype the shorter contiguous sequences.

BLASTn sequence searches and nucleotide alignment to reference sequences obtained from GenBank (previously described) identified only assemblages A and B among the samples. A total of 41/45 sequences were 100% identical to the published sequences for either assemblage A or assemblage B. The remaining four sequences contained a novel single nucleotide deletion at position 162 of the 175 bp 18S rRNA contig, but were otherwise identical to either assemblage A or assemblage B.

Both assemblage A and assemblage B were detected in faecal samples from all three collection periods (Table 2). For the 45 samples genotyped, the majority were identified as assemblage B (34/45, 75.6%), while assemblage A was identified in less than a quarter of samples (11/45, 24.4%) (Table 2). Assemblage B was more commonly found at each of the three collection time periods, in each of the three age groups, and in both males and females (Table 2). Despite an increase in *Giardia* 18S rRNA positivity at collection round 2 (previously described), there was no significant difference in the frequencies of assemblages A and B at collection round 2, compared with round 1 (Table 2).

**Table 2:** *Giardia duodenalis* assemblages identified from children in a remote Indigenous community in the Northern Territory by DNA sequencing of the *Giardia* 18S rRNA locus.

		Faecal samples (n = 45)				Participant data (n = 38)			
		Collection round				Age (years)			
	Gender	Round 1	Round 2	Round 3	Total (%)	0–<5	5–<10	10–<15	Total (%)
Assemblage A	Males	2	3	1	6 (54.5)	4	2	0	6 (54.5)
	Females	1	4	0	5 (45.5)	4	1	0	5 (45.5)
	Total (%)	3 (21.4)	7 (25.0)	1 (33.3)	11 (24.4)	8 (27.6)	3 (42.9)	0 (0.0)	11 (28.9)
Assemblage B	Males	7	10	2	19 (55.9)	12	2	0	14 (51.9)
	Females	4	11	0	15 (44.1)	9	2	2	13 (48.1)
	Total (%)	11 (78.6)	21 (75.0)	2 (66.7)	34 (75.6)	21 (72.4)	4 (57.1)	2 (100.0)	27 (71.1)
Total genotyped		14	28	3	45	29	7	2	38

### Identification of *Giardia* subassemblages by gdh T-RFLP and distribution among samples

A total of 33/87 faecal samples were amplified at the *gdh* locus and genotyping by T-RFLP was successful for 32 samples. *Gdh* T-RFLP analyses identified four different types of genetic profiles present in the samples. Within assemblage A, subassemblage AII was detected in 8/32 (25%) samples (Table 3). Subassemblage AI was not identified. Within assemblage B, two genotypes were detected and both were consistent with the previously described BIII and BIV subassemblages at the *gdh* locus [14]. For consistency, the assemblage B genotypes detected in this study have been labelled as BIII or BIV. Both genotypes were detected separately in samples: BIII (3/32 samples, 9.4%); BIV (12/32 samples, 37.5%) and; as mixed samples of BIII and BIV (9/32 samples, 28.1%) (Table 3). All four infection profiles (AII, BIII, BIV, mixed BIII/BIV) were observed at rounds 1 and 2 of collection (Table 3).

**Table 3:** Distribution of *Giardia duodenalis* assemblages A and B subtypes identified by *gdh* PCR amplification and terminal-RFLP for 32 samples (27 participants) collected from children living in a remote Indigenous community in the Northern Territory.

	Faecal sample data (n = 32)				Participant data (n = 27)					
	Collection round				Age (years)			Gender		
Assemblage A and B subtypes	Round 1	Round 2	Round 3	Total (%)	0–<5	5–<10	10–<15	Males	Females	Total (%)
Subassemblage AII	4	2	2	8 (25.0)	7	1	0	5	3	8 (29.6)
BIII*	1	2	0	3 (9.4)	0	1	2	0	3	3 (11.1)
BIV*	4	8	0	12 (37.5)	10	0	0	7	3	10 (37.0)
Mixed BIII/BIV*	4	5	0	9 (28.1)	5	1	0	3	3	6 (22.2)
Total	13	17	2	32	22	3	2	15	12	27

*BIII and BIV subtypes represent two assemblage B *gdh* genotypes that were identified in this study, and were consistent with the previously described *gdh* BIII and BIV subassemblages [14].

Of the 32 samples genotyped at the *gdh* locus, 28 were also positive by 18S rRNA PCR and of these, 24 were successfully genotyped by 18S rRNA DNA sequencing. Four samples were positive at the *gdh* locus only, but could not be confirmed by 18S rRNA PCR or microscopic examination. Comparison of the *gdh* and 18S rRNA genotyping results showed that assignment of assemblage A and B by *gdh*-T-RFLP and 18S rRNA DNA sequencing was concordant.

### *Giardia* positivity and identification of *Giardia* assemblages among participants that contributed samples at two collection rounds

Thirteen participants contributed samples at two collection rounds (previously described). Of these, 8/9 participants who were 18S rRNA positive at the second collection round were also positive at the first round, and may represent ongoing infections. Of these, 4 participants positive at collection rounds 1 and 2, and one participant positive at collection rounds 2 and 3 were successfully genotyped by 18S rRNA sequencing. All 5 participants were genotyped as assemblage B at both collection rounds where samples were contributed. The *gdh* T-RFLP data showed that 3 of these participants had the same assemblage B subtypes (BIV, mixed BIII/BIV) at both rounds 1 and 2. To determine if the presence of ongoing infections contributed to the significant increase in 18S rRNA positivity at collection round 2, these participants were removed from the round 2 data and the above analyses were repeated. The results showed that the increase in *Giardia* 18S rRNA positive samples in round 2 of collection remained statistically supported (data not shown).

## Discussion

In this study we investigated the prevalence of *Giardia*, and genetic subtypes present in children living in a remote Indigenous community in the Northern Territory. Screening by direct microscopy and 18S rRNA PCR amplification showed that *Giardia* was highly prevalent (66.7%) in faecal samples collected over an 18 month period. The high prevalence of *Giardia* detected in this study is similar to high rates (65%) of *Giardia* previously reported for children living in remote Indigenous communities in Australia [8]. The highest prevalence of *Giardia* was found in 0–<5 year olds, which is similar to that found in low prevalence regions of Australia, where infants aged 0–4 years are the most commonly affected group by sporadic giardiasis [26], [27].

PCR for the 18S rRNA gene proved to be the most sensitive method to detect *Giardia* in the faecal samples. Of the 58 positive samples, 41.4% of these were detected as positive by microscopy, whilst 93.1% were detected as positive using the 18S rRNA PCR. The sensitivity of PCR detection also differed between the two *Giardia* loci that were examined. Differences in detection rates for microscopy and PCR screenings are expected due to intermittent and/or low parasite shedding, DNA polymerase inhibitors in faecal material, and differences in gene copy number for the *gdh* and 18S rRNA loci [11], [28], [29]. Previous studies in remote Indigenous communities of Australia have used microscopy as a preliminary screening tool to determine *Giardia* positivity, and select samples for downstream molecular analyses [17]. The results of the present study, however, demonstrate that a large proportion (58.6%) of positive cases were only detectable by 18S rRNA PCR. Although detection of *Giardia* DNA by PCR is not direct evidence of an established infection, PCR is highly sensitive [30], [31]. Similar screening results have been reported from children living in high prevalence regions of Rwanda, and may reflect low parasite shedding due to constant exposure and chronic infections [28].

*Giardia* was detected in faecal samples from all three collection periods over the 18 month study and high detection rates were maintained over time. A significant increase in PCR positivity, from 45.7% to 70.2%, was detected between collection rounds 1 and 2, and was consistent with an increase in positivity among the 0–<5 year age group over this time. No differences in the frequency of positive cases among males and females, and among assemblage A and B between collection rounds 1 and 2 were detected. Our results suggest an overall increase in PCR positivity during the study; however, further sampling in this community would be required to resolve this. Sample sizes for older age groups were small and participants included in this study were self selected. Participants may have been more likely to provide a faecal sample if gastrointestinal symptoms were present, but additional information pertaining to participant symptoms was not available for this study. It is unknown if PCR positive cases represent established infections, or if the increase in positivity at round 2 represents an increase in *Giardia* infections in this community. In low prevalence regions of Australia, *Giardia* infections fluctuate across demographic groups and with seasonal changes [27]. Factors influencing prevalence in remote Indigenous communities in the Northern Territory are largely unknown due to the listing of *Giardia* as non-notifiable. Our results demonstrate that screening by both microscopy and 18S rRNA PCR is beneficial to accurately determine *Giardia* prevalence, the presence of established infections, and to identify demographic groups within the community that are most at risk from giardiasis.

DNA sequencing analyses of the 18S rRNA gene showed that all *Giardia* cases in this remote community were caused by assemblages A or B. *Giardia duodenalis* assemblage B was most commonly identified overall (75%), at all time points, in all age groups, and in both genders. The predominance of assemblage B concords with other studies from Australia [17], [26]. Further genotyping at the *gdh* locus showed that a diversity of genetic subtypes were present. Within assemblage A, only subassemblage AII was identified. Subassemblage AII is most commonly identified in humans [26] and anthroponotic transmission is likely. Subassemblage AI, the most common human subassemblage found in other animals [13], [26] was not identified in this study. Our results demonstrated that two assemblage B genotypes, consistent with previous *gdh* BIII/BIV descriptions were detected in this community, and both genotypes were detected separately, and as mixed samples. Although designation of isolates to subassemblages BIII or BIV is problematic, an accurate system to classify assemblage B isolates that enables comparison between studies is currently not available. All four types of *Giardia* (subassemblage AII, BIII, BIV, and mixed samples of BIII/BIV) were identified and persisted in the community over a 12 month period.

Detection of mixed genetic variants in 28% of cases is high when compared to data from low prevalence regions of Australia. Studies of sporadic giardiasis in Western Australia and New South Wales have detected mixed assemblage B samples in less than 6% of samples screened [24], [26]. The larger proportion of mixed samples detected in this study may be indicative of environmental contamination and high frequency transmission of different *G. duodenalis* subtypes, and frequent host contact due to overcrowded living conditions, which may contribute to the higher prevalence of mixed infections. The presence of mixed samples can also be explained by nucleotide sequence heterogeneity among assemblage B isolates [11], [32]. The mechanisms that produce sequence heterogeneity are unresolved, but genetic diversity may be more prevalent in highly endemic environments due to increased competition and selective pressures [6].

The results of this study have demonstrated high detection rates of *Giardia* in children living in a remote Indigenous community in the Northern Territory. Gastrointestinal infections remain a significant cause of morbidity in remote Indigenous communities and the burden of infectious diseases extends beyond childhood [3]. The high proportion of positive cases detected in this study among children confirms previous reports from remote Indigenous communities. These results highlight the need for further research to understand the contribution of giardiasis to chronic gastrointestinal disease, and to examine links between PCR positive cases and clinical outcomes.

*Giardia duodenalis* assemblage B dominates transmission in this community. A variety of *G. duodenalis* subtypes persist, and reinfection of children with different genetic variants is possible. The similar frequencies of assemblage A and B in this study to other Australian communities warrants further investigation to assess whether disease dynamics are similar between communities, despite differences in prevalence. In contrast, conditions specific to remote Indigenous communities may enhance mixed or heterogeneous infections.

Knowledge of parasite prevalence, infectious subtypes, and community dynamics that enhance transmission are required to address the continuing burden that gastrointestinal diseases impose on children in remote Indigenous communities.
